# Corticospinal interface to restore voluntary control of joint torque in a paralyzed forearm following spinal cord injury in non-human primates

**DOI:** 10.3389/fnins.2023.1127095

**Published:** 2023-03-07

**Authors:** Kei Obara, Miki Kaneshige, Michiaki Suzuki, Osamu Yokoyama, Toshiki Tazoe, Yukio Nishimura

**Affiliations:** ^1^Neural Prosthetics Project, Tokyo Metropolitan Institute of Medical Science, Tokyo, Japan; ^2^Division of Neural Engineering, Graduate School of Medical and Dental Sciences, Niigata University, Niigata, Japan

**Keywords:** spinal cord injury, closed-loop stimulation, primary motor cortex (M1), spinal stimulation, non-human primates (macaque), corticospinal tract (CST)

## Abstract

The corticospinal tract plays a major role in the control of voluntary limb movements, and its damage impedes voluntary limb control. We investigated the feasibility of closed-loop brain-controlled subdural spinal stimulation through a corticospinal interface for the modulation of wrist torque in the paralyzed forearm of monkeys with spinal cord injury at C4/C5. Subdural spinal stimulation of the preserved cervical enlargement activated multiple muscles on the paralyzed forearm and wrist torque in the range from flexion to ulnar-flexion. The magnitude of the evoked torque could be modulated by changing current intensity. We then employed the corticospinal interface designed to detect the firing rate of an arbitrarily selected “linked neuron” in the forearm territory of the primary motor cortex (M1) and convert it in real time to activity-contingent electrical stimulation of a spinal site caudal to the lesion. Linked neurons showed task-related activity that modulated the magnitude of the evoked torque and the activation of multiple muscles depending on the required torque. Unlinked neurons, which were independent of spinal stimulation and located in the vicinity of the linked neurons, exhibited task-related or -unrelated activity. Thus, monkeys were able to modulate the wrist torque of the paralyzed forearm by modulating the firing rate of M1 neurons including unlinked and linked neurons *via* the corticospinal interface. These results suggest that the corticospinal interface can replace the function of the corticospinal tract after spinal cord injury.

## 1. Introduction

The disruption of descending pathways including the corticospinal tract (CST) results in the loss of connection between the brain and spinal networks and the consequent loss of voluntary motor function. However, the neural circuits located above and below the lesion retain their functions. Electrical stimulation of the spinal cord is a promising method to restore voluntary motor function after the impairment of descending pathways through spinal cord injury (SCI) or stroke. Tonic electrical stimulation of the spinal cord below the lesion has been shown to improve motor function in humans (Minassian et al., 2004; Harkema et al., 2011; Angeli et al., 2014; Lu et al., 2016; Inanici et al., 2018) and animals (Musienko et al., 2009; Kasten et al., 2013; Mondello et al., 2014; Alam et al., 2015) with SCI in which residual descending motor pathways are assumed. Tonic spinal stimulation can raise the excitability of the spared spinal circuits and compensate for the weakened descending commands, which are insufficient for voluntary motor output (Angeli et al., 2014; Rejc et al., 2015; Lu et al., 2016; Gad et al., 2017). Therefore, even uncontrolled open-loop tonic spinal stimulation is useful for the restoration of voluntary motor function in patients with residual descending pathways. In contrast, it is impossible for patients who have completely lost their descending pathways to voluntarily control their paralyzed limb movements by tonic spinal stimulation, even though substantial muscle contractions are produced.

Bypassing the damaged descending pathway using brain-controlled functional electrical stimulation is a promising approach to restore the voluntary control of paralyzed limb movements after the complete loss of descending pathways (Moritz et al., 2008; Pohlmeyer et al., 2009; Ethier et al., 2012; Nishimura et al., 2013; Zimmermann and Jackson, 2014; Bouton et al., 2016; Ajiboye et al., 2017; Kato et al., 2019; Barra et al., 2022). Until recently, the self-execution of paralyzed upper limb movements such as wrist flexion, grasping, and arm retraction has been achieved by brain-controlled functional electrical stimulation of the spinal cord in paralyzed monkeys (Nishimura et al., 2013; Zimmermann and Jackson, 2014; Barra et al., 2022). However, the graded control of force by brain-controlled spinal stimulation has yet to be achieved. Therefore, it is worthwhile assessing the feasibility of brain-controlled spinal stimulation for the modulation of motor output.

Here, we investigated the feasibility of a corticospinal interface through closed-loop brain-controlled subdural spinal stimulation for the modulation of motor output in the paralyzed hand of monkeys with SCI. We found that paralyzed monkeys could modulate motor output such as wrist torque and the activation of multiple forearm muscles by modulating the firing rate of an ensemble of neurons in the primary motor cortex (M1) *via* the corticospinal interface, indicating that a corticospinal interface can compensate for the function of a lesioned CST.

## 2. Materials and methods

### 2.1. Subjects

The experiments were performed using two female macaque monkeys (*Macaca fuscata*: Monkey E, 5.6 kg and Monkey L, 5.0 kg). All experimental procedures were performed in accordance with the guidelines for the Care and Use of Non-human Primates in Neuroscience Research, The Japan Neuroscience Society, and were approved by the Institutional Animal Care and Use Committee of the Tokyo Metropolitan Institute of Medical Science (Approval Nos.: 18035, 19050, and 20-053). The animals were fed regularly with pellets and had free access to water. They were monitored closely and animal welfare was assessed daily or, if necessary, several times a day.

### 2.2. Surgery

All surgical procedures were performed in sterile conditions under general anesthesia induced by ketamine (10 mg/kg, i.m.) plus xylazine (1 mg/kg, i.m.) and maintained with 1–1.5% isoflurane. Atropine (0.12 mg/kg, i.m.), ketoprofen (2 mg/kg, i.m.), maropitant (1 mg/kg, s.c.), and ampicillin (40 mg/kg, i.m.) were administered preoperatively. The depth of anesthesia was confirmed by the pain response. During anesthesia, the animal’s vital signs (respiratory rate, inspiratory CO_2_ concentration, saturation of percutaneous O_2_, heart rate, and body temperature) were monitored carefully. There was no evidence of tachycardia or tachypnea during the surgical procedures nor a major deviation in the heart or respiratory rate in response to noxious stimuli. The absence of reflexive movements to noxious stimuli and corneal reflex was also used to verify the level of anesthesia. Postoperative management consisted of observing the animals until they were completely recovered from the anesthesia, and the administration of ampicillin (40 mg/kg, i.m.), ketoprofen (2.0 mg/kg, i.m.), and dexamethasone (0.825 mg, i.m.).

#### 2.2.1. Cortical array implantation

To record cell activity in M1, we chronically implanted a 96-channel iridium-oxide Utah array (Blackrock Microsystems, Salt Lake City, UT, USA) with an electrode length of 1.5 mm. The array was implanted in the wrist area of the left M1, which was identified by anatomical features and movements evoked by trains of low-intensity electrical stimulation to the cortical surface. The reference electrodes were placed in the subdural space. The ground electrode and connector of the arrays and head-post were anchored to the skull with titanium screws and acrylic cement.

#### 2.2.2. Spinal cord lesioning and electrode implantation on the cervical cord

**FIGURE 1:**
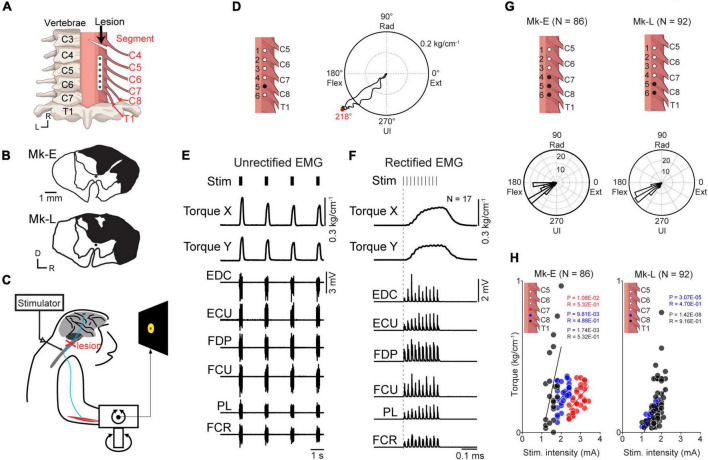
Motor output evoked by subdural spinal stimulation during rest in awake monkeys with SCI. **(A)** A subdural 6-electrode array (platinum) was chronically implanted over the dorsal-lateral aspect of the cervical spinal cord and placed on the C6–T1 segments. A slit at the C4/C5 segment indicates the lesion site. **(B)** Lesion extent (black hatch) at the C4/C5 segment in individual monkeys. **(C)** Subdural spinal stimulation was applied at rest. **(D)** Typical example of average wrist torque trajectory for tonic spinal cord stimulation of C8 (black circle, electrode no. 5). Horizontal and vertical components in this trace correspond to Torque X and Torque Y in panel **(F)**, respectively. Red dot on the torque trajectory represents the maximum magnitude of the evoked torque. **(E)** Raw traces of wrist torque and EMG during subdural spinal stimulation of C8. Stimuli consisting of 10 constant-current biphasic square-wave pulses of 40 Hz with a duration of 0.2 ms and interval of 2 s were delivered through an electrode (Monkey E, post-SCI day 15). **(F)** Stimulus-triggered averages of wrist torque and rectified EMG. The vertical dashed gray lines represent the onset of a stimulus train. **(G)** Population data for the directions of the evoked torque induced by subdural spinal stimulation at rest. Top: black dots on the spinal cord indicate the stimulation sites. Bottom: histograms indicate the directions of the evoked wrist torque. **(H)** The relationship between the magnitude of the evoked wrist torque and stimulus intensity. Colored dots in the figures correspond to spinal stimulus sites. Significant positive correlations between the magnitude of evoked the torque and current intensity were found, shown as solid lines (Pearson correlation coefficient; *P* < 0.05).

Under anesthesia, the border between the C4 and C5 segments was exposed by laminectomy of the C3 and C4 vertebrae, and a transverse opening was made in the dura. A spinal cord lesion was made by transecting the dorsolateral funiculus and dorsal column at the border between C4 and C5 on the right side (Figures 1A, C) under a surgical microscope using fine forceps.

After spinal cord lesioning, incisions were made in the dura mater on the C4 and C7 vertebrae. A 6-channel platinum subdural electrode array, with an electrode diameter of 1 mm and inter-electrode distance of 3 mm (Unique Medical Corporation, Tokyo, Japan), was implanted on the right side of the cervical enlargement (C6–T1). The array was slid into the subdural space from the incision site at the C7 vertebra, and placed over the dorsal-lateral aspect of the C6–T1 segments, where the dorsal rootlets are located (Figure 1A). The incision on the dura was covered with gel foam and the laminectomy was closed with acrylic cement. A silver plate (3 mm × 2 mm) was used as a reference electrode and placed on the T1 vertebra. The bundle of electrode wires covered with silicon tubing was glued with dental acrylic to bone screws placed in the T1 dorsal process and subcutaneously routed to the skull and its connector was mounted with acrylic resin. The skin and back muscle incisions were sutured with silk or nylon threads, respectively.

#### 2.2.3. Implantation of microwires on forelimb muscles

Electromyography (EMG) wires were surgically implanted in the right arm and hand muscles. The target muscles were identified by anatomical features and movements evoked by trains of low-intensity electrical stimulation. Bipolar, multi-stranded stainless-steel wires (AS631, Cooner Wire Company, Chatsworth, CA, USA) were sutured into each muscle and routed subcutaneously to the skull, and their connectors (MCP-12-SS; Omnetics, Minneapolis, MN, USA) were anchored to the skull. The EMG electrodes were implanted in the following 11 muscles. Four digit muscles: flexor digitorum superficialis (FDS), extensor digitorum communis (EDC), flexor digitorum profundus (FDP), and extensor digitorum 4 and 5 (ED45); five wrist muscles: flexor carpi radialis (FCR), palmaris longus (PL), flexor carpi ulnaris (FCU), extensor carpi ulnaris (ECU), and extensor carpi radialis (ECR); and two elbow muscles: biceps brachii (BB) and brachioradialis (BR).

### 2.3. Outline of the corticospinal interface

**FIGURE 2:**
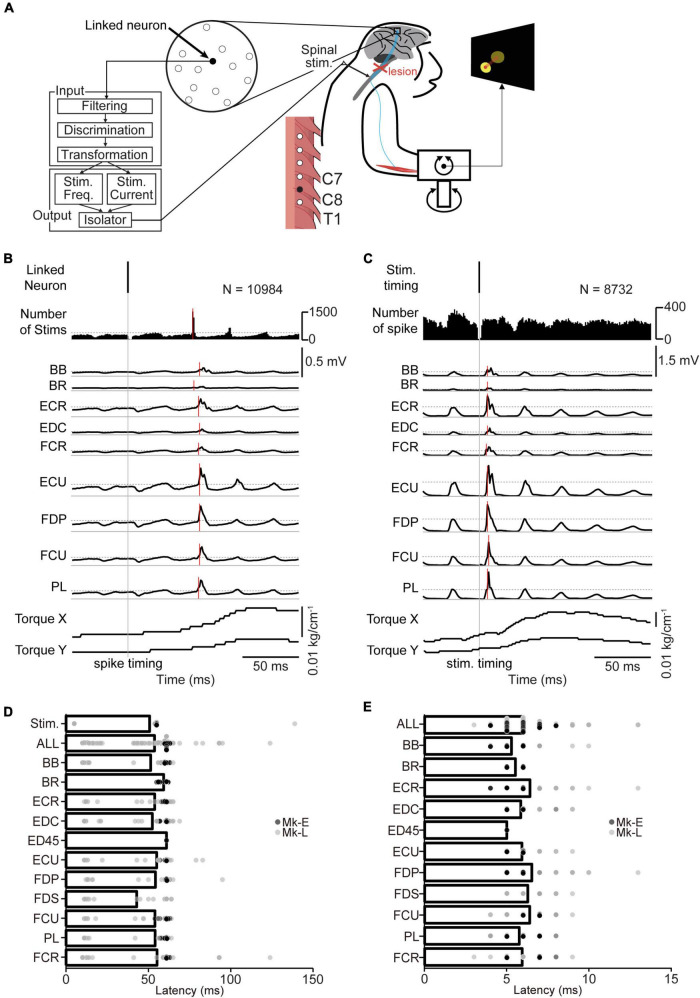
The corticospinal interface. **(A)** Design of the corticospinal interface that translates the activity of a linked neuron to electrical stimulation of the cervical enlargement. **(B)** Typical examples of spike-triggered averages (SpTAs) of rectified EMGs and torque while a linked neuron was connected to the spinal site *via* the corticospinal interface. Traces were aligned to the spike timing of a linked neuron (vertical gray solid line). Vertical red horizontal line indicates the onset. The horizontal gray dot lines in number of stims and EMGs traces represent + 3 SDs of SpTAs calculated during the baseline period (50–0 ms preceding the spike trigger pulse), respectively. From the 1st row: spike of the linked neuron (1st row), spinal stimulation (2nd row), rectified EMG traces (3rd–11th rows), and wrist torques (12th and 13th rows). **(C)** Typical examples of stimulus-triggered averages (StTAs) of rectified EMGs and torque while a linked neuron was connected to the spinal site *via* the corticospinal interface. Traces are aligned to the timing of spinal stimulation (vertical gray solid line). Vertical red solid lines indicate the onset. The horizontal gray dot lines in EMG traces represent + 3 SDs of StTAs calculated during the baseline period (50–0 ms preceding the stimulus trigger pulse), respectively. From the 1st row: spinal stimulation (1st row), spike of the linked neuron (2nd row), EMG traces (3rd–11th rows), and wrist torques (12th and 13th rows). The data were obtained from Monkey E. **(D)** The onset latency of the spinal stimulation and rectified EMGs from the spike of a linked neuron [ALL: *N* = 564, PL and ECR: *N* = 62, FDS: *N* = 24, FDP and EDC: *N* = 61, ED45: *N* = 2, BR: *N* = 40, others: *N* = 63 (Monkey E, ALL: *N* = 360, FDS and ED45: *N* = 2, FDP and EDC: *N* = 38, others: *N* = 40; Monkey L, ALL: *N* = 204, PL, FDS, and ECR: *N* = 22, ED45 and BR: *N* = 0, others: *N* = 23)]. Bars indicate mean values. **(E)** The onset latency of the rectified EMGs from the spinal stimulation [ALL: *N* = 567, FDS: *N* = 25, FDP and EDC: *N* = 61, ED45: *N* = 2, BR: *N* = 40, others: *N* = 63 (Monkey E, ALL: *N* = 360, FDS and ED45: *N* = 2, FDP and EDC: *N* = 38, others: *N* = 40; Monkey L, ALL: *N* = 207, ED45 and BR: *N* = 0, others: N = 23)]. Bars indicate mean values.

**FIGURE 3:**
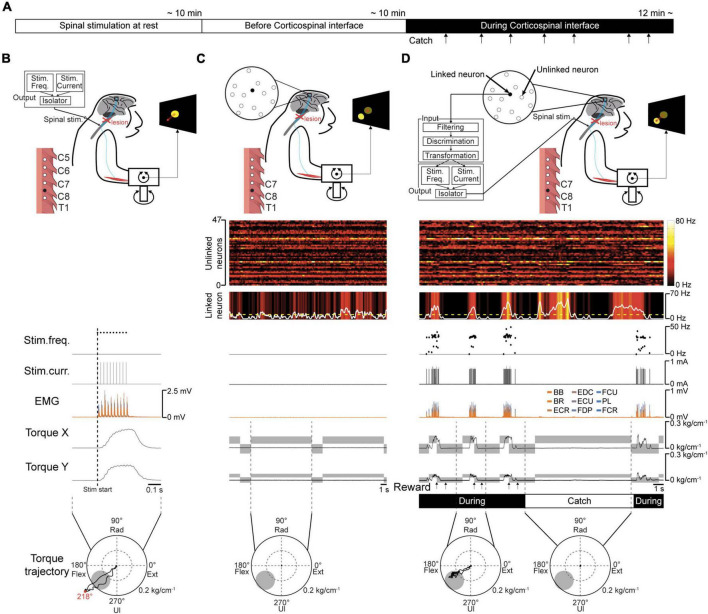
Volitional control of a paralyzed forearm using the corticospinal interface. **(A)** Experimental procedure. First, an experiment of “Spinal stimulation at rest” was conducted to confirm the direction and magnitude of the evoked wrist torque induced by tonic spinal stimulation at rest. Next, the monkeys performed the torque-tracking task without the corticospinal interface as an experiment of “Before corticospinal interface”. Subsequently, a linked neuron was connected to the spinal site *via* the interface, which was called an experiment of “During corticospinal interface”. Catch trials (upward arrows) were interleaved at random intervals. **(B)** An example of an experiment of “Spinal stimulation at rest”. EMG and wrist torque were produced by stimulation of C8 at 1.8 mA and 40 Hz. The peripheral target position (gray circle in two-dimensional plot of wrist torque) was set in the same direction as the evoked torque and at a location at which half of the maximum magnitude of evoked torque was required. **(C)** An example of an experiment of “Before corticospinal interface”. The monkeys controlled the position of a cursor (red circle) using wrist torque to acquire targets (yellow circle) displayed on the screen. The activity of a single neuron (linked neuron, black) in the hand area of M1 was detected in order to utilize its neuronal activity as an input source for controlling the stimulation of a single spinal site (black) in the next experiment of “During corticospinal interface”. **(D)** An example of an experiment of “During corticospinal interface”, including three successful trials when the corticospinal interface was on (During, 8th row) and one catch trial when it was switched off (Catch, 8th row). The modulation of 48 neurons (1st and 2nd rows) was detected through the Utah array in M1 and the activity of a single neuron (linked neuron, 2nd row) was selected from them as the input signal for controlling stimulus frequency (3rd row) and intensity (4th row) *via* the corticospinal interface. Stimulation frequency and current were determined according to the firing rate of the linked neuron above a stimulation threshold (yellow dashed line in the 2nd row). The gray rectangles in the wrist torque traces (6th and 7th rows) represent the peripheral and center targets. The arrows at the bottom indicate successful trial completion and the delivery timing of the juice reward (7th row).

To regain volitional control of the paralyzed forearm, a corticospinal interface that connected an arbitrarily selected neuron in M1 and a spinal site caudal to the SCI site was used (Figure 2). A two- or three-graded torque-tracking task was used to evaluate the motor function of the right wrist. One experimental session consisted of three experiments (Figure 3A) as follows. To determine a peripheral target location for voluntary torque control, the direction and magnitude of the evoked wrist torque was confirmed first by applying current to an arbitrarily selected electrode on the cervical enlargement while the monkeys were at rest (Figures 1, 3B, “Spinal stimulation at rest”). Next, to investigate the firing pattern of M1 cells before applying the corticospinal interface, data were obtained without the corticospinal interface (Figure 3C, “Before corticospinal interface trials”). Finally, the corticospinal interface was then connected from an arbitrarily selected neuron in M1 to a spinal site located caudal to the SCI (Figure 3D, “During corticospinal interface trials”). The corticospinal interface was designed to detect the firing rate of an arbitrarily selected neuron and convert it in real time to activity-contingent electrical stimulation of a spinal site located caudally to the SCI. To verify that the monkeys could not acquire the peripheral target through volitional muscle contractions, it was sometimes turned off during a catch trial (“Catch” in Figure 3D, “During catch trials”).

In total, both monkeys completed 63 sessions, using 11 different pairs of neurons in M1 and spinal sites [Table 1, Monkey E, *N* = 40 sessions (7 sessions included catch trials); Monkey L, *N* = 23 sessions (1 session included catch trials)].

**TABLE 1:** Summary of the experiments.

Monkey	Post-SCI day	Cortical linked neuron	Spinal site (Figure 1D)	Stim. intensity (mA)		Number of targets
				*I*_0_	*I*_Max_	
E	8	ch26	6	1.50	1.60	2
	9	ch26	6	1.50	1.60	2
	9	ch26	6	1.50	1.60	2
	10	ch26	6	1.30	1.40	2
	11	ch26	6	1.60	1.70	2
	12	ch26	6	1.70	1.80	2
	13	ch26	5	1.70	1.80	2
	14	ch26	5	1.70	1.80	2
	15	ch26	5	1.70	1.80	2
	16	ch26	5	1.70	1.80	3
	17	ch26	5	1.70	1.80	3
	17	ch26	5	1.90	2.00	3
	18	ch26	5	1.70	1.80	3
	18	ch26	5	1.70	1.80	3
	19	ch26	5	2.10	2.20	3
	20	ch26	5	2.10	2.20	3
	21	ch26	5	1.90	2.00	3
	22	ch26	5	2.30	2.40	3
	23	ch26	5	2.30	2.40	3
	24	ch26	5	2.30	2.40	3
	25	ch26	5	2.30	2.40	3
	26	ch26	5	2.30	2.40	3
	27	ch26	5	2.00	2.10	2
	27	ch26	5	2.30	2.40	2
	28	ch26	5	2.30	2.40	2
	29	ch26	5	2.20	2.30	3
	30	ch26	5	2.20	2.30	3
	31	ch26	4	2.90	3.00	3
	34	ch26	4	3.10	3.20	2
	35	ch26	4	3.10	3.20	2
	37	ch26	4	3.10	3.20	2
	38	ch26	4	3.10	3.20	2
	39	ch96	4	3.10	3.60	2
	40	ch78	4	2.50	3.00	2
	41	ch78	4	2.30	2.90	2
	42	ch78	4	2.30	2.50	2
	42	ch78	4	2.50	2.60	2
	43	ch78	4	2.50	2.60	2
	44	ch78	4	2.50	2.60	2
	46	ch78	4	2.50	2.60	2
L	2	ch42	6	1.40	1.50	2
	3	ch42	6	1.40	1.70	2
	3	ch38	6	1.40	1.50	2
	6	ch14	6	1.10	1.80	2
	9	ch72	6	1.50	1.66	2
	10	ch72	6	1.80	2.02	2
	11	ch72	6	1.42	2.00	2
	12	ch72	6	1.36	1.60	2
	13	ch72	6	1.44	1.60	2
	15	ch72	6	1.60	1.90	2
	18	ch72	6	1.50	2.00	2
	20	ch62	6	1.50	1.60	2
	21	ch62	6	1.50	1.70	2
	22	ch62	6	1.46	1.60	2
	23	ch62	6	1.50	1.60	2
	24	ch62	6	1.44	1.66	2
	25	ch62	6	1.60	1.90	2
	26	ch62	6	1.70	1.90	2
	28	ch62	5	1.48	1.58	3
	30	ch62	5	1.10	1.36	3
	31	ch62	5	1.10	1.38	3
	32	ch62	5	1.10	1.26	3
	33	ch62	5	1.10	1.30	3
Total	11 pairs				63 sessions	

Electrode 1 was located on the rostral cervical cord (C6 rostral), and electrode 6 was located on the caudal cervical cord (T1 rostral). In the target column, 2 and 3 indicate a two-graded task and three-graded task, respectively.

#### 2.3.1. Investigation of the relationship between spinal stimulation and motor output

To determine the stimulus parameters for the corticospinal interface, “Spinal stimulation at rest” tests were conducted at the beginning of each session (Figure 3B). While the right upper limb was fixed in an experimental apparatus recording two-dimensional wrist isometric torque (Figure 1A), subdural spinal stimuli consisting of 10 constant-current, biphasic square-wave pulses (each pulse 0.2 ms in duration) were delivered at 40 Hz through a single electrode using a stimulator (ULI-100; Unique Medical Corporation, Tokyo, Japan) targeting an arbitrarily selected electrode on the cervical enlargement. Stimulus trains were delivered 3–225 times with an interval of 2,000 ms (Figures 1E, F). The direction and magnitude of the evoked wrist torque was measured at a stimulus intensity between 1.0 and 3.4 mA (Figures 1D, G, H).

#### 2.3.2. Real-time corticospinal interface

To achieve a corticospinal interface that sends voluntary commands to the preserved spinal site by bypassing the spinal lesion, the firing rate of an arbitrarily selected neuron (linked neuron) in M1 was converted into stimulus pulses, and electrical stimulation was delivered through an arbitrarily selected electrode on the cervical enlargement. The corticospinal interface was accomplished using a computer interface that was designed to detect the action potentials of the linked neuron specifically using a template-matching algorithm (Blackrock Microsystems, Salt Lake City, UT, USA) and convert them in real time into a stimulus current and frequency that were dependent on the firing rate of the linked M1 cell. The moving averaged firing rate (50-ms time window) of the linked neuron had a proportional relationship with the stimulation current and frequency; thus, the monkeys could voluntarily co-modulate the current and frequency of the electrical stimuli by changing the firing rate of the linked neuron (Figure 2A).

If the averaged firing rate of the linked neuron [*X* (Hz)] was above the stimulus threshold [*X*_*th*_ (Hz)], the stimulus frequency [*f* (Hz)] and current [*I* (mA)] were modulated by the following equations:


$$f=f_{0}+\frac{f_{\text{g}}}{X_{\text{th}}}\left(X-X_{\text{th}}\right),\left(f_{0}\leq f\leq f_{\text{Max}}\right)$$

where *f*_0_ = initial stimulus frequency when *X* (Hz) was above *X*_*th*_ (Hz), *f*_*g*_ = gain of the stimulus frequency, *f*_*Max*_ = maximum stimulus frequency (Hz).


$$I=I_{0}+\frac{I_{\text{g}}}{X_{\text{th}}}\left(X-X_{\text{th}}\right),\left(I_{0}\leq I\leq I_{\text{Max}}\right)$$

where *I*_0_ = initial stimulus current, *I*_*g*_ = gain of the stimulus current, *I*_*Max*_ = maximum stimulus current (mA).

In both monkeys, the stimulus parameters were determined based on the results obtained in the testing periods “Spinal stimulation at rest” and “Before corticospinal interface” as follow: *X*_*th*_, 10–60 Hz; *f*_0_, 30 Hz; *f*_*g*_, 5 Hz; *f*_*Max*_, 40 Hz; *I*_0_, 1.10–3.10 mA; *I*_*g*_, 0.02 mA; *I*_*Max*_, 1.26–3.60 mA. Each parameter had to meet the following criteria: *X*_*th*_, higher than the average firing rate of the linked neuron during the “Before corticospinal interface” period; *f*_0_ and *I*_0_, the initial stimulus frequency and intensity that did not allow the monkeys to reach the peripheral target (see section “2.4. Behavioral task”); *f*_*g*_ and *I*_*g*_, the gains of stimulus frequency and intensity that could induce a smooth movement trajectory, respectively; *f*_*Max*_ and *I*_*Max*_, the maximum stimulus frequency and intensity that generated an overshoot of the peripheral targets (see section “2.4. Behavioral task”).

The initial stimulus current (*I*_0_), and maximum stimulus current (*I*_*Max*_) were sometimes adjusted to maintain a consistent relationship between wrist torque and the firing rate of the linked neurons.

### 2.4. Behavioral task

Before SCI, each monkey was trained to control the position of a cursor on a video monitor with isometric wrist torque (torque-tracking task) and to acquire targets displayed on the screen as described elsewhere (Nishimura et al., 2013; Kato et al., 2019; Kaneshige et al., 2022). In this task, the movement direction of the cursor on the screen coincided with the direction of wrist torque (Figure 3). Behavioral experiments started after the monkey’s performance reached 10 trials/min for 10 consecutive sessions prior to SCI without the corticospinal interface. Trials were initiated by entering the center target and holding for a period of 800 ms. The “Go” cue (appearance of a peripheral target) was provided after the hold period. After SCI, the peripheral target position was set on the way of the evoked torque trajectory confirmed in the “Spinal stimulation at rest” testing period, so that the wrist torque required to hit the target was set at 25–70% (gray circle in the bottom panels of Figure 3) of the evoked peak torque (red dot in the bottom panel of Figure 3B). The “End” cue (appearance of a center target) was provided after a peripheral hold period of 300–400 ms. A liquid reward was provided after a successful reach to each target and a center hold period of 500 ms. The monkeys were required to clear the hold criterion within 10 s. When the hold criterion was met or the 10-s period was not achieved, the next target was presented, either immediately or after a reward period (Inter-trial interval: 1 s). The monkeys participated in a total of 63 torque-tracking task sessions with the corticospinal interface (Monkey E, 40 sessions; Monkey L, 23 sessions). In several sessions (Monkey E, 16/40 sessions; Monkey L, 5/23 sessions), the monkeys performed a three-graded torque-tracking task in which peripheral targets appeared at two different positions (i.e., different magnitudes of wrist torque in the same direction were required to perform the task successfully). In the three-graded torque-tracking task, trials in which a peripheral target was located close to the center target (“Weak” torque trials) required the production of 60% of the wrist torque required in trials in which a peripheral target was located farther from the center target (“Strong” torque trials). The timing of when the cursor entered the peripheral targets (“In”) was defined as the last time the cursor entered the peripheral target after the “Go” cue during a successful trial (Figure 7).

### 2.5. Data collection

A 96-channel array was connected to a multi-channel amplifier. Neural signals were recorded at a sampling rate of 30 kHz and a bandpass filter was applied at 250–7,500 Hz. EMG signals were amplified using a multichannel amplifier (AB-611J; Nihon Kohden, Tokyo, Japan) at a gain of ×100 and bandpass filtered at 50–3,000 Hz. EMG signals, wrist torque (flexion-extension and ulnar-radial directions), task parameters such as target positions, and the timing of trial events were recorded simultaneously with the neural signal using a Cerebus multichannel data acquisition system (Blackrock Microsystems, Salt Lake City, UT, USA) at a sampling rate of 10 kHz. All recorded signals were down-sampled to 1 kHz for offline analysis.

### 2.6. Data analysis

#### 2.6.1. Evoked muscle activity and wrist torque

To minimize the effect of artifact contamination by spinal electrical stimulation on EMG recordings, the raw EMG data from 2 ms before to 2 ms after stimulus timing were removed, and the remaining data were analyzed.

The stimulus- or spike-triggered averages of rectified EMG and wrist torque data were compiled (Figures 1F, 2B, C). The magnitude and angle of wrist torque were measured when the average wrist torque induced by spinal stimulation reached the maximum value (red dot in right panel of Figure 1D). To investigate the relationship between the current intensity of spinal stimulation and the magnitude of the evoked torque, Pearson correlation coefficients were computed between them for each spinal site (Figure 1H).

Mean baseline activity and standard deviation were measured from rectified EMG traces in the period from 50 to 0 ms preceding the trigger pulse. The onset latency of muscle activation or stimulation of the biggest response was detected as greater than 3 standard deviations from the mean baseline (Figures 2B, C).

#### 2.6.2. Neuronal activity

Spikes from single M1 units were sorted using the Offline Sorter software package (Plexon, Dallas, TX, USA) by projecting waveforms into principal component space and identifying isolated clusters, and spike timings were smoothed (window: 200 ms) and down-sampled from 30 to 1 kHz for offline analysis. Neuronal activity was analyzed separately in neurons linked to spinal stimulation (linked neurons) and others (unlinked neurons). For a fair comparison between before and during the corticospinal interface condition, data from the same number of trials (9–55 trials) before and during the corticospinal interface condition were analyzed. Data during the corticospinal interface condition were extracted from a peak performance period in the first 10 min. The data in the catch trials were extracted from the entire corticospinal interface condition.

Unlinked neurons were classified into task-related neurons and task-unrelated neurons (“unrelated neurons”) as follows. The average firing rate of each neuron was calculated in a 400-ms period around two task events: before the Go cue (Figures 5A, 7A: –500 to –100 ms relative to peripheral target appearance) and after the Go cue (Figure 5A: 100 to 500 ms relative to peripheral target appearance, Figure 7A: –200 to 200 ms relative to the timing of “In”). A neuron was defined as “task-related” if there was a significant difference in its average firing rate between before and after the Go cue. Then, the task-related neurons were classified into “increased neurons” and “decreased neurons” as follows. An increased neuron was defined by a significant increase of its firing rate after the Go cue relative to before the Go cue, and a decreased neuron was defined by a significant decrease of its firing rate after the Go cue relative to before the Go cue (Figures 5A, 7A).

To examine changes in the activity of unlinked neurons in representative sessions (Figures 5A, 7A), the firing rates of the unlinked neurons were *z*-scored using the firing rates during a 400-ms period (500–100 ms before the Go cue).

#### 2.6.3. Task-related modulation

To examine the changes of activity before and after peripheral target appearance, the modulation depths (MDs) of neural activity, EMG, and torque were calculated. MD was defined as the difference in the average firing rate of M1 cells, rectified EMG, and wrist torque between before the Go cue (Figures 5A, 7A: –500 to –100 ms relative to peripheral target appearance) and after the Go cue (Figure 5A: 100 to 500 ms relative to peripheral target appearance; Figure 7A: –200 to 200 ms relative to the timing of “In”) in each session.

#### 2.6.4. Task performance

Task performance was defined as the maximum number of successful trials/min in each condition.

#### 2.6.5. Statistical analysis

**FIGURE 4:**
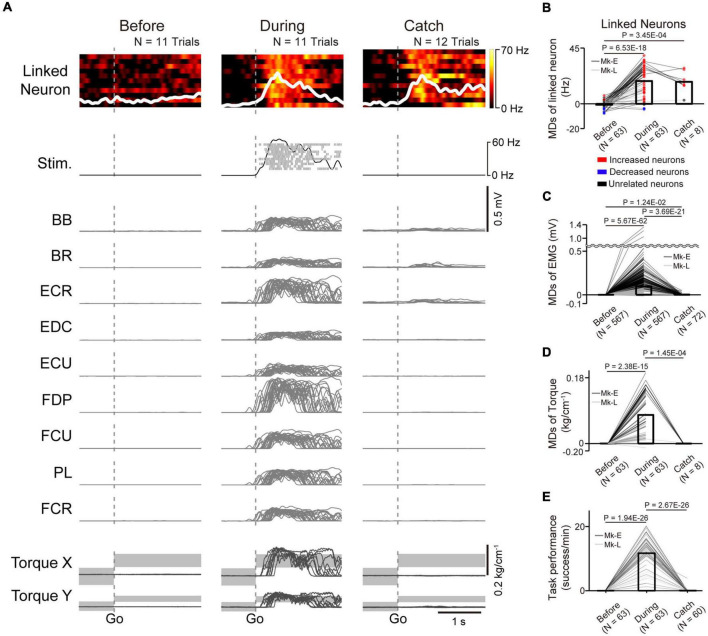
Task-related modulation of linked neurons, EMG, and torque. **(A)** Examples of the firing rate in individual trials (heatmap) and the average firing rate (white trace) of a linked neuron (1st row), spinal stimulation (2nd row), EMG of the forelimb (3rd–11th rows), and wrist torque (12th and 13th rows) before (left panel) and during the corticospinal interface (center panel) and catch trials (right panel). Plots are aligned to the timing of target appearance, indicated by the vertical dotted lines. The gray-shaded rectangles in the bottom traces represent the target range of the required torque for a successful trial. MDs of the firing rates of linked neurons **(B)**, EMG **(C)**, and wrist torque **(D)** before (left bar) and during the corticospinal interface (center bar) and catch trials (right bar) [*N* = 63 sessions before and during corticospinal interface, 8 sessions during catch trials (Monkey E, *N* = 40 sessions before and during corticospinal interface, 7 sessions during catch trials; Monkey L, *N* = 23 sessions before and during corticospinal interface, 1 session during catch trials)]. Bars indicate mean values. Black horizontal lines represent significant differences (*P* < 1.67 × 10^–2^ by paired *t*-test with Bonferroni’s correction). Colors of the circles represent the neuron types sorted in each condition (i.e., before and during corticospinal interface and catch trials). Sessions with at least nine trials in each condition were included in the analysis. **(E)** Task performance before and during the corticospinal interface trials and during the catch trials [*N* = 63 sessions before and during corticospinal interface, 60 sessions during catch trials (Monkey E, *N* = 40 sessions before and during corticospinal interface, 38 sessions during catch trials; Monkey L, *N* = 23 sessions before and during corticospinal interface, 22 sessions during catch trials)]. Bars indicate mean values. Black horizontal lines represent significant differences (*P* < 1.67 × 10^–2^ by paired *t*-test with Bonferroni’s correction for *post hoc* multiple comparisons). Sessions with at least one trial in each condition were included in the analysis.

To determine whether there were statistically significant differences in the MDs of the firing rate of M1 cells, rectified EMG, wrist torque, and task performance before and during the corticospinal interface (two- and three-graded tasks) and during the catch trials (Figures 4B–E, 6A–I, 7B–E, 8A–I), a paired *t*-test with Bonferroni’s correction was performed.

**FIGURE 5:**
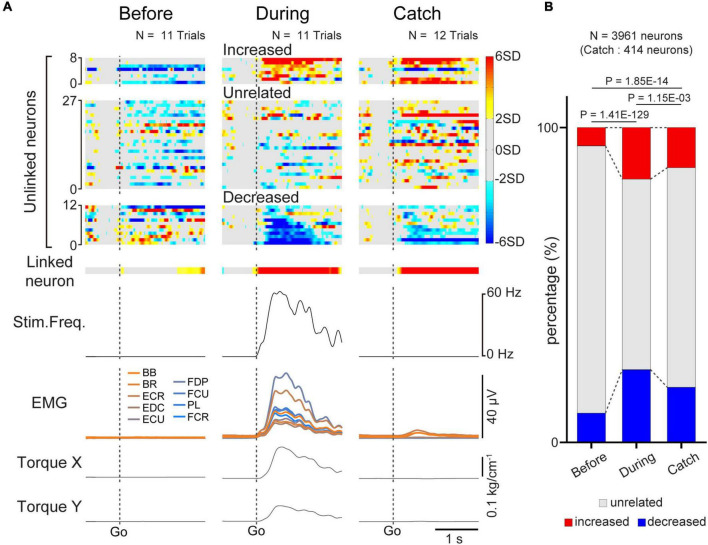
Task-related modulation of unlinked neurons. **(A)** Examples of average firing rate of M1 cells (1st and 2nd rows), stimulus frequency (3rd row), EMG of the forelimb (4th row), and wrist torque (5th and 6th rows) before (left panel) and during the corticospinal interface trials (center panel) and during the catch trials (right panel). *Z*-scored firing rates of unlinked (1st row) neurons and linked (2nd row) neurons are shown. Unlinked neurons are sorted into “increased”, “decreased”, and “unrelated” neurons according to activity during the corticospinal interface sessions. Plots are aligned to the timing of target appearance (“Go”), indicated by the vertical dotted lines. **(B)** The percentage of the types of unlinked neurons (red: “increased” neuron, black: “unrelated” neuron, blue: “decreased” neuron) before and during the corticospinal interface and catch trials. Black horizontal lines represent significant differences (*P* < 1.67 × 10^–2^ by Chi-squared test with Bonferroni’s correction for *post hoc* multiple comparisons).

**FIGURE 6:**
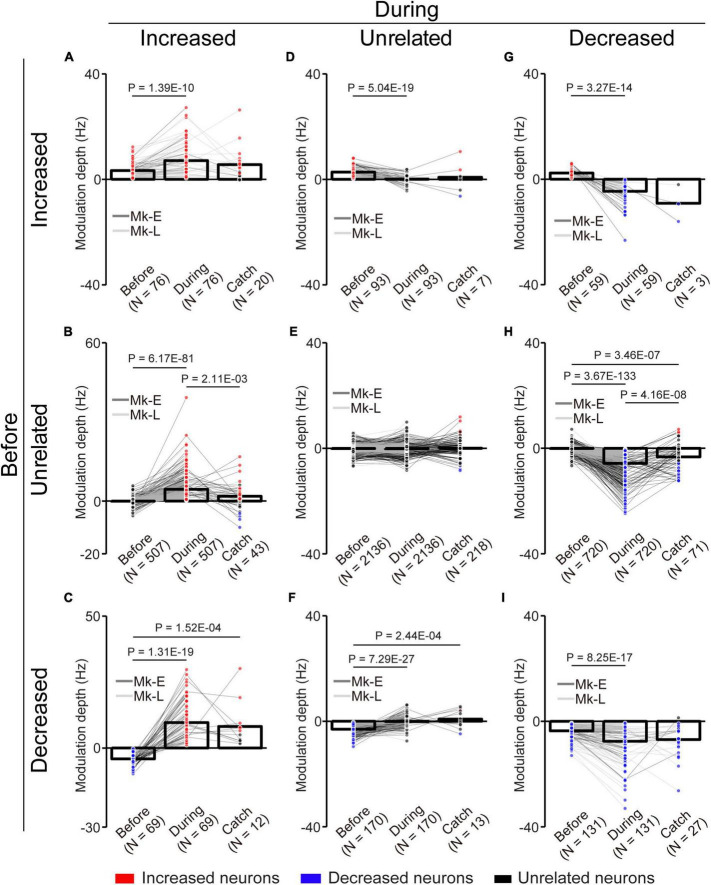
Change of the MDs of unlinked neurons with the corticospinal interface. **(A)** Neurons maintained their properties as “increased” type before and during the corticospinal interface trials. **(B)** Neurons changed their properties from “unrelated” to “increased” type. **(C)** Neurons changed their properties from “decreased” to “increased” type. **(D)** Neurons changed their properties from “increased” to “unrelated” type. **(E)** Neurons maintained their properties as “unrelated” type. **(F)** Neurons changed their properties from “decreased” to “unrelated” type. **(G)** Neurons changed their properties from “increased” to “decreased” type. **(H)** Neurons changed their properties from “unrelated” to “decreased” type. **(I)** Neurons maintained their properties as “decreased” type. Bars and circles indicate the MDs of mean values and individual neurons, respectively. Colors (red: increased neuron, black: unrelated neuron, blue: decreased neuron) of the circles represent the neuron type sorted in each condition (i.e., experiments of before and during the corticospinal interface and catch trials). Black horizontal lines represent significant differences (*P* < 1.67 × 10^–2^ by paired *t*-test with Bonferroni’s correction for *post hoc* multiple comparisons). Experiments with at least nine trials were included in each condition.

**FIGURE 7:**
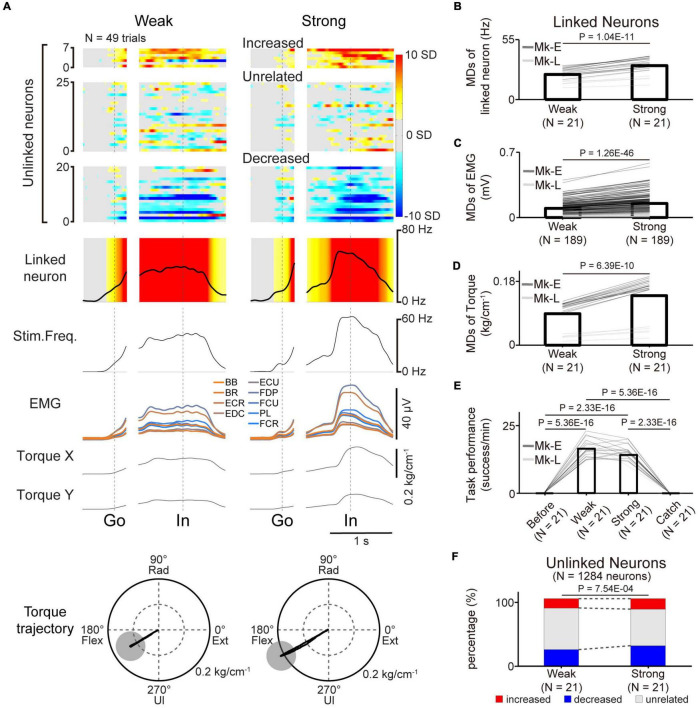
Volitional control of a paralyzed forearm during a three-graded torque-tracking task with the corticospinal interface. **(A)** Examples of the average M1 firing rate (1st and 2nd rows), stimulus frequency (3rd row), EMG of the forelimb (4th row), and wrist torque (5th and 6th rows) in the weak torque trials (left panel) or strong torque trials (right panel) for a representative session. Heatmap indicates *Z*-scored firing rates of unlinked and linked neurons. Plots are aligned when the peripheral target appeared (“Go”) or when the cursor entered the peripheral target (“In”), indicated by the vertical dotted lines. Torque trajectories are two-dimensional plots of the average wrist torque in the weak torque trials (left) and strong torque trials (right). The gray circles represent the targets of peripheral wrist torque. **(B–F)** Change of M1 neurons, EMG, wrist torque, and task performance during the three-graded torque-tracking task [*N* = 21 sessions in the weak and strong torque trials (Monkey E, *N* = 16 sessions; Monkey L, *N* = 5 sessions)]. Black horizontal lines represent significant differences. Bars in panels **(B–F)** indicate mean values. **(B)** According to the increase of the required torque, the MDs of the linked neurons increased (*P* < 0.05 by paired *t*-test). **(C,D)** Statistical analysis: *P* < 0.05 by paired *t*-test. **(E)** Task performance during the corticospinal interface trials was significantly higher than before the corticospinal interface trials and during the catch trials (*P* < 8.33 × 10^–3^ by paired *t*-test with Bonferroni’s correction for *post hoc* multiple comparisons). **(F)** The percentage of increased and decreased neurons was increased in the strong torque trials (*P* < 0.05 by Chi-squared test).

**FIGURE 8:**
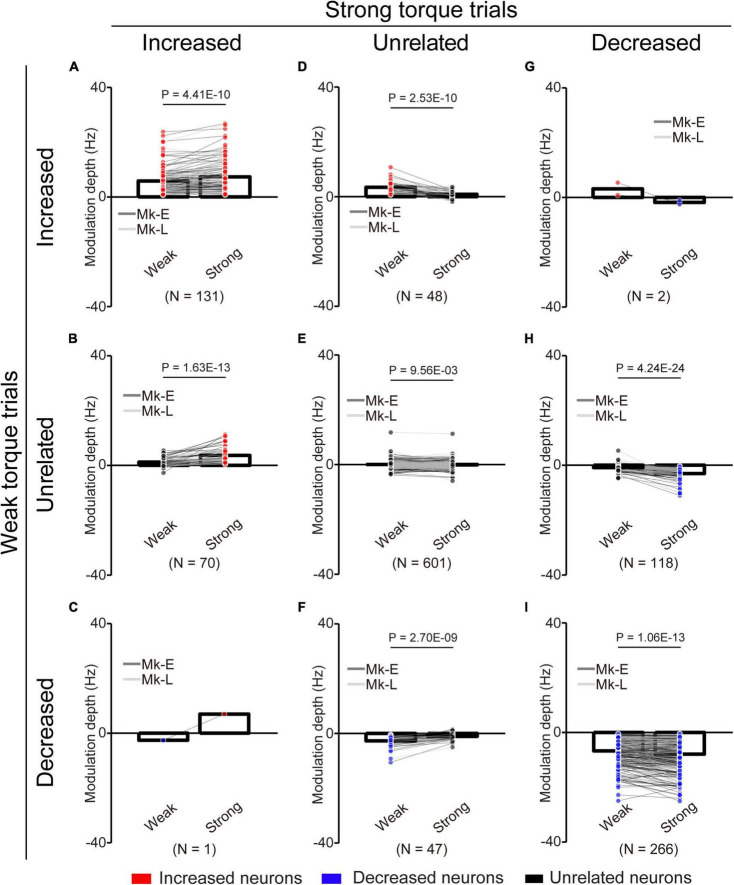
Change of the MDs of unlinked neurons at different torque requirements. **(A)** Neurons maintained their properties as “increased” type throughout the experiments. **(B)** Neurons changed their properties from “unrelated” to “increased” type. **(C)** Neurons changed their properties from “decreased” to “increased” type. **(D)** Neurons changed their properties from “increased” to “unrelated” type. **(E)** Neurons maintained their properties as “unrelated” type. **(F)** Neurons changed their properties from “decreased” to “unrelated” type. **(G)** Neurons changed their properties from “increased” to “decreased” type. **(H)** Neurons changed their properties from “unrelated” to “decreased” type. **(I)** Neurons maintained their properties as “decreased” type. Bars and circles indicate the MDs of mean values and individual neurons, respectively. Colors (red: increased neuron, black: unrelated neuron, blue: decreased neuron) of the circles represent the neuron types sorted in each condition (i.e., before and during the corticospinal interface and catch trials). Black horizontal lines represent significant differences (*P* < 0.05 by paired *t*-test with Bonferroni’s correction). Experiments with at least nine trials were included in each condition.

**FIGURE 9:**
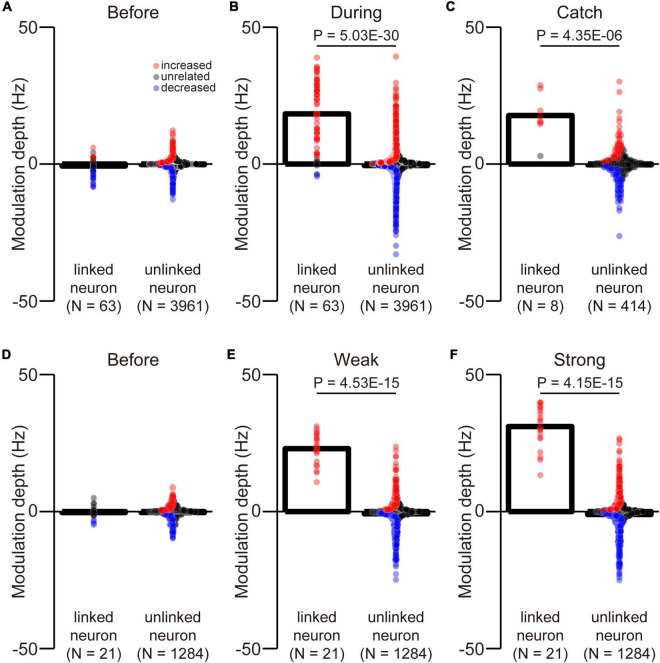
Difference between the MDs of linked and unlinked neurons. The MDs of linked and unlinked neurons before **(A)** and during the corticospinal interface trials **(B)** and during catch trials **(C)**. The MDs of linked and unlinked neurons before the corticospinal interface trials **(D)** and during weak **(E)** and strong trials **(F)**. Bars and circles indicate the MDs of mean values and individual neurons, respectively. Colors (red: increased neuron, black: unrelated neuron, blue: decreased neuron) of the circles represent the neuron types sorted in each condition (i.e., before and during the corticospinal interface and catch trials). Black horizontal lines represent significant differences (*P* < 0.05 by Wilcoxon rank-sum test). Experiments with at least nine trials were included in each condition.

To determine whether there were statistically significant differences in the MDs of the firing rate of the linked and unlinked neurons before and during the corticospinal interface (two- and three-graded tasks) and during the catch trials (Figure 9), the Wilcoxon rank-sum test was performed.

The classification of unlinked neurons into “task-related neurons”, “task-unrelated neurons”, “increased neurons”, and “decreased neurons” was based on the *P*-value of a paired *t*-test.

To compare the percentages of the type of unlinked neurons before and during the corticospinal interface and between the weak and strong torque trials, a Chi-squared test was used (Figures 5B, 7F).

Statistical significance was considered at *P* < 0.05, unless otherwise noted.

All statistical analyses were performed with MATLAB 2014a and 2021a statistical tool box (MathWorks, Inc., Natick, MA, USA) and R (version 4.1.1; R Foundation for Statistical Computing, Vienna, Austria).

### 2.7. Confirmation of lesion extent

At the end of all experiments, the monkeys were anesthetized deeply with an overdose of sodium pentobarbital (50 mg/kg, i.v.) and perfused transcardially with 0.1 M phosphate-buffered saline (pH 7.4), followed by 10% formaldehyde in 0.1 M phosphate buffer (pH 7.4). The perfused spinal cord was removed and immersed successively in 10, 20, and 30% sucrose in 0.1 M phosphate buffer (pH 7.3). The specimens were cut serially into coronal sections of 50-μm thickness on a freezing microtome, and every 5th section was mounted on a gelatin-coated glass slide and Nissl-stained with 0.5% cresyl violet. Photomicrographs of the spinal cord lesion were captured. The extent of the lesion was defined by the area of gliosis.

## 3. Results

### 3.1. A primate spinal lesion model

Two macaque monkeys were subjected to unilateral SCI that was limited to the border between the C4 and C5 segments on the right side (Figures 1A, B). The lesion was extended into the lateral funiculus and dorsal column including a substantial portion of the descending and ascending pathways (Figure 1B). Immediately after lesioning, Monkey E displayed hemiplegia on the ipsilesional side. No apparent movement of the forearms, including the finger and wrist joints, was observed, but there was weak muscle activity at the elbow and shoulder joints on the ipsilesional side. The lower extremity showed a nearly complete motor deficit on the ipsilesional side. Monkey L displayed a nearly complete motor deficit of the upper and lower extremities on both sides. Since the animals did not respond to noxious mechanical stimulation of body parts on the lesioned side, somatosensory functions appeared to be impaired on the lesioned side in both animals. Experiments in Monkeys E and L were performed until post-SCI day 45 and 33, respectively. Neither animal showed an improvement of the voluntary control of the fingers and wrist joint throughout the experimental period.

### 3.2. Evoked wrist torque by subdural spinal stimulation during rest

To confirm the effect of subdural spinal stimulation on muscle activity of the forearm and wrist torque, tonic spinal stimuli were delivered at various current intensities from an electrode on the cervical enlargement (C6–T1) in two monkeys with SCI. Subdural spinal stimuli consisting of 10 constant-currents at 40 Hz were delivered through a single electrode while the monkeys were not required to produce any wrist torque to hold a cursor in a resting position of a center target (Figure 1). Figures 1D–F shows typical examples of the wrist torque and EMG responses induced by subdural spinal stimulation of C8 at 1.8 mA (electrode no. 5, Monkey E, post-SCI day 14). Spinal stimulation induced responses in multiple muscles and wrist torque (Figures 1E, F). The magnitude and direction of the evoked torque were 0.27 kg/cm^–1^ and ulnar-flexion (218°, right panel in Figure 1D), respectively. Figure 1G shows the population data for the direction of the evoked torque. Tonic spinal stimuli at various current intensities (Monkey E, 1.2–3.4 mA; Monkey L, 1.0–2.2 mA) at the caudal region of the cervical enlargement (black circles in the top panels of Figure 1G) induced wrist torque in the direction of flexion to ulnar-flexion (Monkey E, 179–243°; Monkey L, 193–260°). The magnitude of the evoked torque was positively correlated with current intensity [Figure 1H, Monkey E: electrode 4 (red), *R* = 0.53, *P* = 1.08 × 10^–2^; electrode 5 (blue), *R* = 0.49, *P* = 9.81 × 10^–3^; electrode 6 (black), *R* = 0.53, *P* = 1.74 × 10^–3^; Monkey L: electrode 5 (blue), *R* = 0.47, *P* = 3.07 × 10^–5^; electrode 6 (black), *R* = 0.92, *P* = 1.42 × 10^–8^]. These results demonstrated that subdural spinal stimulation of the preserved cervical enlargement induced the activation of multiple forearm muscles and wrist torque of the paralyzed forearm in the range from flexion to ulnar-flexion. We also found that the magnitude of the evoked torque could be controlled by changing current intensity.

### 3.3. Volitional control of the paralyzed forearm *via* a corticospinal interface

To regain volitional control of the paralyzed forearm, we employed a corticospinal interface that connected an arbitrarily selected neuron in M1 (linked neuron) and a spinal site for bridging the SCI site. The firing rate of an arbitrarily selected linked neuron was converted into stimulus pulses, and electrical stimulation was delivered through an arbitrarily selected electrode on the cervical enlargement (Figure 2A). Figure 2B shows the latencies of spinal stimulation and muscle activation from the action potentials of a linked neuron. The average latency of spinal stimulation was 50.7 ± 19.4 ms [Figure 2D, 363–19,258 spikes in 63 sessions during the corticospinal interface trials (Monkey E, *N* = 2,694–19,258 spikes; Monkey L, *N* = 363–12,461 spikes), Monkey E, 55.0 ± 0.16 ms; Monkey L, 43.3 ± 31.2 ms]. The average latency of evoked muscle activity was 53.7 ± 17.5 ms (Figure 2D, Monkey E, 60.6 ± 1.41 ms; Monkey L, 41.6 ± 24.7 ms). The latencies of muscle activation in proximal muscles such as the BB and BR were similar to those of distal muscles such as the EDC, ED45, and FDS [Figure 2D, PL and ECR: *N* = 62, FDS: *N* = 24, FDP and EDC: *N* = 61, ED45: *N* = 2, BR: *N* = 40, others: *N* = 63 (Monkey E, FDS and ED45: *N* = 2, FDP and EDC: *N* = 38, others: *N* = 40; Monkey L, PL, FDS, and ECR: *N* = 22, ED45 and BR: *N* = 0, others: *N* = 23)].

We also investigated the latency of muscle activation from spinal stimulation (Figure 2C). The average latency of muscle activation from spinal stimulation was 5.98 ± 1.19 ms [Figure 2E, 345–13,130 spikes in 63 sessions during the corticospinal interface trials (Monkey E, *N* = 1,956–13,130 spikes; Monkey L, *N* = 345–9,753 spikes), Monkey E, 5.80 ± 0.80 ms; Monkey L, 6.30 ± 1.61 ms]. The latencies of muscle activation in proximal muscles were similar to those of distal muscles [Figure 2E, FDS: *N* = 25, FDP and EDC: *N* = 61, ED45: *N* = 2, BR: *N* = 40, others: *N* = 63 (Monkey E, FDS and ED45: *N* = 2, FDP and EDC: *N* = 38, others: *N* = 40; Monkey L, ED45 and BR: *N* = 0, others: *N* = 23)].

To determine a peripheral target location for voluntary torque control, the direction and magnitude of evoked wrist torque were confirmed by injecting current to an arbitrarily selected spinal site while the monkeys were at rest. The representative example in Figure 3B shows the trajectory of wrist torque induced by subdural electrical stimulation of C8 at 1.8 mA. The peripheral target location was set on the evoked trajectory and at half the maximum torque value induced by the tested current (gray circle in the bottom panels in Figures 3B–D). Therefore, the monkeys were required to regulate the torque output of the paralyzed forearm by modulating the firing rate of the linked neuron that controls the current and frequency of spinal stimulation to acquire the target.

To investigate the firing pattern of M1 cells before applying the corticospinal interface, data were obtained in its absence. The firing patterns of most M1 neurons, forelimb muscle activity, and wrist torque showed no apparent changes related to the task requirements (Figure 3C).

The corticospinal interface was then connected from a linked neuron to a spinal site located caudally to the SCI. The corticospinal interface was designed to detect the firing rate of an arbitrarily selected “linked neuron” and convert it in real time to activity-contingent electrical stimulation to a spinal site located caudally to the SCI. The current intensity and frequency applied to the spinal site were proportional to the firing rate of the linked neuron. The monkeys could regulate the current intensity and frequency of the electrical stimulation by altering the firing rate of the linked neuron (Figure 3D); thus, they could control the activity of the paralyzed wrist muscles and the magnitude of wrist torque, leading to repeated target acquisition. To confirm the feasibility of the corticospinal interface, it was turned off during catch trials (“Catch” in Figure 3D). During the catch trials, the monkeys continued to increase the firing rate of the linked neuron; however, they were unable to acquire the peripheral target due to paralysis, indicating that the corticospinal interface was necessary for the voluntary control of wrist torque.

To investigate how monkeys with SCI utilized the corticospinal interface, we investigated the activity of linked neurons and paralyzed muscles and wrist torque. Figure 4A shows a typical example of the firing pattern of a linked neuron, muscle activity, and wrist torque before and during the corticospinal interface and during the catch trials (Monkey E, post-SCI day 15, electrode: 5, *I*_0_: 1.7 mA, *I*_*Max*_: 1.8 mA, *I*_*g*_: 0.01 mA, *f*_0_: 30 Hz, *f*_*Max*_: 40 Hz, *f*_*g*_: 5 Hz, pulse width: 0.2 ms). The firing rate of the linked neuron did not show remarkable modulation before the corticospinal interface trials (left panel in Figure 4A), while it showed task-related modulation that increased after peripheral target appearance during the corticospinal interface and catch trials (center and right panels in Figure 4A). The frequency of spinal stimulation, EMG, and wrist torque were also co-modulated with the firing rate of the linked neuron during the corticospinal interface trials (center panels in Figure 4A), whereas negligible muscle activity and no apparent wrist torque were produced before the corticospinal interface and during the catch trials (left and right panels in Figure 4A). The MDs of the linked neurons during the corticospinal interface and catch trials were significantly increased compared to before the corticospinal interface trials (Figure 4B, paired *t*-test with Bonferroni’s correction: *P*_*before vs.during*_ = 6.53 × 10^–18^, *P*_*before vs.* during *catch trials*_ = 3.45 × 10^–4^). Similarly, the MDs of EMG (Figure 4C) and torque (Figure 4D) during the corticospinal interface trials were also significantly increased compared to before the corticospinal interface trials (Figure 4C, paired *t*-test with Bonferroni’s correction: *P*_*before vs.* during_ = 5.67 × 10^–62^; Figure 4D, paired *t*-test with Bonferroni’s correction: *P*_*before vs.* during_ = 2.38 × 10^–15^). However, the MDs of EMG (Figure 4C) and torque (Figure 4D) during the catch trials were significantly decreased compared to during the corticospinal interface trials (Figure 4C, paired *t*-test with Bonferroni’s correction: *P*_*during vs.* during catch trials_ = 3.69 × 10^–21^; Figure 4D, paired *t*-test with Bonferroni’s correction: *P*_*during vs.* during catch trials_ = 1.45 × 10^–4^) due to the absence of spinal stimulation, and the monkeys failed to acquire the peripheral target (Figure 3D, right panel in Figure 4E).

In total, both monkeys performed the experiments in 63 sessions, using 11 different pairs of neurons in M1 and spinal sites [Table 1, Monkey E, *N* = 40 sessions (catch: 7 sessions of those included in the catch trials); Monkey L, *N* = 23 sessions (catch: 1 session of those included in the catch trials)]. The monkeys reached peak performance at 6.19 ± 2.99 min (Monkey E, 7.15 ± 2.69 min; Monkey L, 4.52 ± 2.78 min) in the first 10 min during the corticospinal interface. The average peak task performance was significantly lower with the corticospinal interface after SCI [11.70 ± 5.31 trials/min, (Monkey E, 13.18 ± 4.73 trials/min, *N* = 40 sessions; Monkey L, 10.23 ± 5.82 trials/min, *N* = 23 sessions)] than without the corticospinal interface before SCI [19.34 ± 1.63 trials/min, (Monkey E, 17.78 ± 0.29 trials/min, *N* = 10 sessions; Monkey L, 20.91 ± 0.25 trials/min, *N* = 10 sessions), unpaired *t*-test: *P*_*before SCI vs. after SCI*_ = 5.52 × 10^–8^], but was significantly higher than before the corticospinal interface and during the catch trials after SCI (Figure 4E, paired *t*-test with Bonferroni’s correction: *P*_*before vs.* during_ = 1.94 × 10^–26^, *P*_*before vs.* catch trials_ = 0.321, *P*_*during vs.* catch trials_ = 2.67 × 10^–26^). These results suggest that the corticospinal interface was essential for the voluntary control of the wrist torque of the paralyzed forearm.

### 3.4. Task-related modulation of unlinked neurons during the corticospinal interface

Since we used a multi-channel electrode array, which enabled the recording of assemblies of M1 neurons, we investigated how unlinked neurons, which were not connected to the interface, modulated their activity in response to the corticospinal interface. Figure 5A shows a typical example of the task-related modulation of linked and unlinked neurons before and during the corticospinal interface and during the catch trials (Monkey E, post-SCI day 15, Electrode: 5, *I*_0_: 1.7 mA, *I*_*Max*_: 1.8 mA, *I*_*g*_: 0.01 mA, *f*_0_: 30 Hz, *f*_*Max*_: 40 Hz, *f*_*g*_: 5 Hz, pulse width: 0.2 ms). Before the corticospinal interface trials, most of the unlinked neurons did not show task-related modulation of their activity, as for a linked neuron (left panel in Figure 5A). Conversely, during the corticospinal interface trials, many unlinked neurons exhibited task-related modulation of their activity. We found two types of unlinked neurons exhibiting task-related activity: neurons that increased their firing rate and neurons that decreased their firing rate in response to the required torque (center panel in Figure 5A). During the catch trials, task-related modulation in the unlinked neurons was similar to the activity during the corticospinal interface trials. Although spinal stimulation was not applied in the catch trials, only the proximal arm muscles showed small changes in their activity. However, the wrist muscles did not show any activity, so the monkeys failed to generate wrist torque (right panel in Figure 5A).

To characterize the change in the activity of the unlinked neurons, they were classified into “task-related” and “task-unrelated” neurons (unrelated neurons, middle panels of the heatmap in Figure 5A) (see section “2. Materials and methods”). The task-related neurons were further classified into “increased” (top panels of the heatmap in Figure 5A) neurons and “decreased” (bottom panels of the heatmap in Figure 5A) neurons, which showed increased and decreased activity in response to the task, respectively (Figure 5). Although the majority were “task-unrelated” unlinked neurons before the corticospinal interface trials, the percentage of “task-unrelated” unlinked neurons decreased during the corticospinal interface and catch trials, indicating that the firing pattern of “task-unrelated” unlinked neurons changed to that of “task-related” neurons [Figure 5B, 3,961 neurons in 63 sessions before and during corticospinal interface trials (Monkey E, *N* = 1,846 neurons; Monkey L, *N* = 2,115 neurons), 414 neurons in eight sessions in catch trials (Monkey E, *N* = 312 neurons; Monkey L, *N* = 102 neurons), Chi-squared test: χ^2^ = 593.15, *P* = 4.70 × 10^–127^]. In addition, the MDs of neuronal firing in the “increased” (Figure 6A, paired *t*-test with Bonferroni’s correction: *P*_*before vs.* during_ = 1.39 × 10^–10^, *P*_*before vs. during catch trials*_ = 1.54 × 10^–1^, *P*_*during vs. during catch trials*_ = 3.21 × 10^–1^; Figure 6B, paired *t*-test with Bonferroni’s correction: *P*_*before vs. during*_ = 6.17 × 10^–81^, *P*_*before vs. during catch trials*_ = 2.47 × 10^–2^, *P*_*during vs. during catch trials*_ = 2.11 × 10^–3^; Figure 6C, paired *t*-test with Bonferroni’s correction: *P*_*before vs. during*_ = 1.31 × 10^–19^, *P*_*before vs. during catch trials*_ = 1.52 × 10^–4^, *P*_*during vs. during catch trials*_ = 4.33 × 10^–1^) and “decreased” (Figure 6G, paired *t*-test with Bonferroni’s correction: *P*_*before vs. during*_ = 3.27 × 10^–14^, *P*_*before vs. during catch trials*_ = 1.16 × 10^–1^, *P*_*during vs. during catch trials*_ = 2.23 × 10^–1^; Figure 6H, paired *t*-test with Bonferroni’s correction: *P*_*before vs. during*_ = 3.67 × 10^–133^, *P*_*before vs. during catch trial*_ = 3.46 × 10^–7^, *P*_*during vs. during catch trials*_ = 4.16 × 10^–8^; Figure 6I, paired *t*-test with Bonferroni’s correction: *P*_*before vs. during*_ = 8.25 × 10^–17^, *P*_*before vs. during catch trials*_ = 3.12 × 10^–2^, *P*_*during vs. during catch trials*_ = 5.83 × 10^–1^) neurons were greater during the corticospinal interface trials than before them. Conversely, “unrelated” neurons during the corticospinal interface trials showed a smaller change of the MDs or maintained their characteristics in different trial types (Figure 6D, paired *t*-test with Bonferroni’s correction: *P*_*before vs. during*_ = 5.04 × 10^–19^, *P*_*before vs. during catch trials*_ = 1.44 × 10^–1^, *P*_*during vs. during catch trials*_ = 9.39 × 10^–1^; Figure 6E, paired *t*-test with Bonferroni’s correction: *P*_*before vs. during*_ = 9.74 × 10^–1^, *P*_*before vs. during catch trials*_ = 2.19 × 10^–2^, *P*_*during vs. during catch trials*_ = 3.42 × 10^–1^; Figure 6F, paired *t*-test with Bonferroni’s correction: *P*_*before vs. during*_ = 7.29 × 10^–27^, *P*_*before vs. during catch trials*_ = 2.44 × 10^–4^, *P*_*during vs. during catch trials*_ = 6.20 × 10^–1^). Thus, a subgroup of “unlinked” neurons also responded to the corticospinal interface as well as “linked” neurons. Conversely, the MDs during the catch trials tended to be smaller than those during the corticospinal interface trials (Catch in Figures 5A, 6B, H).

### 3.5. Modulation of the torque of the paralyzed hand *via* a corticospinal interface

The results demonstrated that the linked neurons showed task-related modulation *via* the corticospinal interface, and this modulation contributed to success in the torque-tracking task. However, it was not clear whether this modulation was caused by the monkeys simply aiming for a certain firing rate of a linked neuron or if they understood the relationship between the evoked torque and the target and modulated the firing rate of a linked neuron as needed. To investigate whether the monkeys recognized this relationship, we conducted a three-graded torque-tracking task by setting targets that required the monkeys to generate “Weak” torque, “Strong” torque, or no torque. Figure 7A illustrates a typical example of neuronal activity, EMG, and wrist torque when targets requiring “Weak” and “Strong” torque were presented. The monkeys successfully completed the task by adjusting wrist torque to the required amount for each target (Monkey E, post-SCI day 16, Electrode: 5, *I*_0_: 1.7 mA, *I*_*Max*_: 1.8 mA, *I*_*g*_: 0.01 mA, *f*_0_: 30 Hz, *f*_*Max*_: 40 Hz, *f*_*g*_: 5 Hz, pulse width: 0.2 ms). The linked neurons varied their firing rates according to the required magnitude of wrist torque. The MDs of the linked neurons in the “Strong” torque trials were significantly greater than those of the “Weak” torque trials (Figure 7B, paired *t*-test: *P* = 1.04 × 10^–11^), and the MDs of EMG and torque in the “Strong” torque trials were also significantly greater than those of the “Weak” torque trials (EMG in Figure 7C, paired *t*-test: *P* = 1.26 × 10^–46^; wrist torque in Figure 7D, paired *t*-test: *P* = 6.39 × 10^–10^). There was no significant difference in task performance between the “Weak” and “Strong” torque trials (Figure 7E, paired *t*-test with Bonferroni’s correction: *P*_*before vs.* in weak torque trials_ = 5.36 × 10^–16^, *P*_*before vs. in strong torque trials*_ = 2.33 × 10^–16^, *P*_*in weak torque trials vs. in strong torque trials*_ = 5.05 × 10^–2^, *P*_*in weak torque trials vs. during catch trials*_ = 5.36 × 10^–16^, *P*_*in strong* torque trials vs. *during catch trials*_ = 2.33 × 10^–16^, *P*_*before vs. during catch trials*_ = 1). Thus, monkeys with SCI were able to grade wrist torque voluntarily *via* the corticospinal interface, suggesting that they understood the relationship between the amount of evoked torque required to control the cursor and the target location and modulated the firing rate of linked neurons as needed.

The firing rates of a subgroup of unlinked neurons were modulated in the same manner as the linked neurons depending on the required magnitude of wrist torque (Figure 7A). To investigate whether the unlinked neurons changed their characteristics according to the required torque, the percentage of characteristic combinations (“increased”, “decreased”, or “unrelated”) in the “Weak” and “Strong” torque trials was calculated [Figure 7F, total in both monkeys: 21 sessions (Monkey E, *N* = 16 sessions; Monkey L, *N* = 5 sessions), 1,284 neurons (Monkey E, *N* = 768 neurons; Monkey L, *N* = 516 neurons)]. The majority of neurons maintained their characteristics at different torques, although the percentage of “task-unrelated” unlinked neurons was decreased in the strong trials, indicating that some “task-unrelated” unlinked neurons changed their firing characteristics to “task-related” neurons with either “increased” or “decreased” characteristics (Figure 7F, Chi-squared test: χ^2^ = 14.381, *P* = 7.54 × 10^–4^).

To clarify the possibility that even if neurons maintained their characteristics (“increased” or “decreased”), they changed their MDs, we compared the MDs of the unlinked neurons between the “Weak” and “Strong” torque trials (Figure 8). Neurons that consistently showed “increased” (Figure 8A, paired *t*-test: *P* = 4.41 × 10^–10^), “unrelated” (Figure 8E, paired *t*-test: *P* = 9.56 × 10^–3^), and “decreased” (Figure 8I, paired *t*-test: *P* = 1.06 × 10^–13^) characteristics in the “Weak” and “Strong” torque trials had significantly greater MDs in the “Strong” torque trials than in the “Weak” torque trials. Thus, the unlinked neurons also modulated their activity depending on the required magnitude of wrist torque.

### 3.6. Difference in modulation between linked and unlinked neurons

We selected an arbitrary linked neuron from among an ensemble of M1 neurons. However, it was unclear whether they had similar properties as unlinked neurons. To investigate selection bias, we compared the MDs of linked and unlinked neurons before and during the corticospinal interface and during catch trials. There was no difference in the MDs between the linked and unlinked neurons before the corticospinal interface (Figure 9A, Wilcoxon rank-sum test: *P* = 9.75 × 10^–2^; Figure 9D, Wilcoxon rank-sum test: *P* = 6.84 × 10^–1^). The results indicate that the selection of neurons was unbiased. However, during the corticospinal interface and catch trials, there were significant differences between the MDs of linked and unlinked neurons (During corticospinal interface, Figure 9B, Wilcoxon rank-sum test: *P* = 5.03 × 10^–30^; During catch trials, Figure 9C, Wilcoxon rank-sum test: *P* = 4.35 × 10^–6^). These results were also significantly different in the weak and strong trials (Weak, Figure 9E, Wilcoxon rank-sum test: *P* = 4.53 × 10^–15^; Strong, Figure 9F, Wilcoxon rank-sum test: *P* = 4.15 × 10^–15^).

## 4. Discussion

The aim of this study was to investigate the feasibility of a corticospinal interface for the graded control of wrist torque of a paralyzed hand in monkeys with SCI at C4/C5. The current intensity of subdural spinal stimulation on the preserved cervical enlargement could modulate the magnitude of activation of paralyzed forearm muscles and wrist torque. To send voluntary commands to the preserved spinal site by bypassing the spinal lesion, we employed a corticospinal interface that connected an arbitrarily selected neuron in M1 and a spinal site. The corticospinal interface modulated the current intensity and frequency of spinal cord stimulation in proportion to the firing rate of the linked neuron. Paralyzed monkeys were able to modulate torque output at the wrist joint by modulating the firing rate of M1 neurons *via* the corticospinal interface, indicating that the interface compensated for the function of the lesioned CST.

### 4.1. Current intensity controls the magnitude of torque output, but not its direction

Intact animals chiefly employ ordered motor unit recruitment and rate coding to modulate muscle force output. As the level of contraction increases, additional motor units are recruited, and the firing rates of motor units increase (Adrian and Bronk, 1929). Our results showed that the magnitude of the evoked wrist torque changed according to the stimulus current and was positively correlated with current intensity (Figure 1H), indicating that current change was associated with the number and firing rate of the recruited motor units. Furthermore, as we applied repetitive stimulation at 40 Hz (as shown in Figure 1F), temporal summation of the membrane potential of spinal neurons and the resulting torque output also contributed to the production of stronger torque. These types of temporal and spatial summation mechanisms play a role in modulating torque output.

Since the subdural array covered the dorsal-lateral aspect of the cervical enlargement beneath the dorsal root and dorsolateral funiculus (Figure 1B), which contains corticospinal and rubrospinal tracts, electrical currents are likely to first drive the afferent fibers adjacent to the stimulation site, indicating that a major component of the stimulus effect could be driven by the spinal reflex *via* large-diameter and low-threshold afferent fibers. As stimulus current increases, it might drive the intersegmental spinal circuitry and evoke the activation of multiple muscles. In addition, stimulation might activate descending tracts located in the dorsolateral funiculus, such as the corticospinal and rubrospinal tracts, directly innervating the spinal circuits in the cervical enlargement. Further higher currents, which induced a larger magnitude of wrist torque, might spread to the ventral aspect of the spinal cord and lead to the direct activation of motor axons. Thus, increasing current of subdural spinal stimulation supposedly permits gradually recruitment of smaller to larger motoneurons, which in turn, achieves gradient control of torque output.

Motor output from spinal stimulation has been examined extensively in anaesthetized conditions, showing only excitatory effects for epidural spinal stimulation (Greiner et al., 2021) and intraspinal microstimulation (Saigal et al., 2004; Moritz et al., 2007; Zimmermann et al., 2011). In awake animals, spinal stimulation induces excitatory and/or inhibitory effects on muscle activity during voluntary movements (Nishimura et al., 2013; Kato et al., 2020; Kaneshige et al., 2022). The magnitude of this activation depends on stimulation intensity (Kato et al., 2020; Kaneshige et al., 2022). However, the effect of current intensity on motor output from spinal stimulation in awake injured animals is unknown. Our results from awake monkeys with SCI showed that inhibitory effects were unobservable due to the lack of background activity of the paralyzed forearm muscles. However, subdural spinal cord stimulation induced muscle activity in the paralyzed forearm (Figures 1E, F). These results indicate that the excitability of the spinal motoneuron pool is too low to observe the effect of inhibitory spinal interneurons on motor output. This result was consistent with those obtained under anaesthetized conditions in previous studies (Kato et al., 2020), indicating that the excitability of spinal motoneurons in SCI is quite low due to the lack of descending inputs.

In daily life, we are required to control movements in a variety of directions, but unfortunately, the present results in SCI animals with paralyzed forearm showed that spinal stimulation of C7–T1 at rest could only induce torque in a limited range of directions. Spinal stimulation at rest activated multiple muscles including flexor, extensor, ulnar, and radial muscles about the wrist joint, while the directions of the evoked torque responses were limited in the ulnar-flexion direction, irrespective of current intensity (Figure 1G). This result corresponds with our previous study demonstrating that subdural spinal stimulation at higher currents evokes stereotypical torque responses in the ulnar-flexion direction during voluntary torque production (Kaneshige et al., 2022). This finding might be due to the large proportion of spinal interneurons affecting flexor muscles (Perlmutter et al., 1998), a biomechanical interaction between bones, ligaments, and musculotendon units for forearm movements (Razavian et al., 2022), and the fact that the number and volume of wrist flexor and ulnar muscles are greater than those of antagonist muscles (wrist radial and extensor muscles), so that the evoked torque is limited in the ulnar-flexion direction.

### 4.2. Voluntarily controlled motor output through the corticospinal interface

As mentioned above, voluntary contraction of skeletal muscles is controlled by two mechanisms: one changes the number of active motor units and the other changes the firing rate of individual motor units. Both mechanisms are regulated by commands from descending pathways including the corticospinal neurons in the motor cortex. One is the number of active descending neurons and the other is the firing rate of the activated descending neurons. The corticospinal interface in the present study was designed to emulate these processes and the anatomical connections of the CST, which connects the M1 to the spinal cord. The interface was programmed to utilize the firing rate of a single M1 neuron and convert it in real time to activity-contingent electrical stimulation of a spinal site. The stimulation current and frequency applied to a spinal site were proportional to the firing rate of a single neuron (Figures 2A, 3D). In the corticospinal interface, modulation of the stimulation current and frequency by a linked neuron is assumed to alter the number and firing rate of corticospinal neurons which associate with the linked neuron, respectively. The increased current might increase the excitability of the spinal circuits that recruit more spinal motoneurons, as well as increase the firing rate of active motoneurons. The increased frequency may also increase the excitability of the spinal circuits *via* temporal and spatial summation of membrane potentials in spinal neurons, thus facilitating recruitment and the rate-coding process. As a result, the task-related activity of the linked neurons in M1 modulated the magnitude of the evoked torque and the activation of multiple muscles depending on the required magnitude of wrist torque (Figures 4, 5, 7).

Descending commands generated in the motor cortex for controlling voluntary limb movements activate spinal motoneurons and interneurons. The functional loss of limb control in individuals with SCI or stroke can be caused by the interruption of corticospinal pathways originating from the motor cortex, although the neural circuits located above and below the lesion remain functional. A substantial portion of corticospinal pathways are derived from M1 (Toyoshima and Sakai, 1982; He et al., 1993; Usuda et al., 2022). Numerous studies have shown that the neural activity in M1 represents the level of muscle activity (Fetz and Cheney, 1980; Cheney et al., 1985; Buys et al., 1986; Lemon et al., 1986), joint torque (Evarts, 1968; Kakei et al., 1999), and force (Cheney and Fetz, 1980; Sergio and Kalaska, 2003). Thus, M1 is the most appropriate cortical source of the input signal controlling stimulation to the preserved spinal cord for the control of muscle activation and joint torque. Indeed, the activity of a single neuron (Moritz et al., 2008; Zimmermann and Jackson, 2014) or an ensemble of neurons (Pohlmeyer et al., 2009; Ethier et al., 2012; Nishimura et al., 2013; Bouton et al., 2016; Ajiboye et al., 2017; Kato et al., 2019; Barra et al., 2022) in M1 can be used as a signal to control the stimulation parameters to determine the contraction level of paralyzed muscles. The motor cortex contains corticospinal neurons that project directly to the spinal cord and neurons that project to other subcortical nuclei or the cerebral cortex. A corticospinal neuron controls the activity of multiple target muscles (Fetz and Cheney, 1979; Cheney et al., 1982). Regardless of the original function or anatomical connectivity of the linked neuron, the corticospinal interface enabled the linked neuron to innervate the spinal circuits as an artificial corticospinal neuron. Thus, the monkeys were able to modulate stimulation of the preserved spinal cord and wrist torque of the paralyzed hand by modulating the firing rate of the artificial corticospinal neuron. This result suggests the corticospinal interface replaced the function of the CST after SCI. However, the innate CST and corticospinal interface do not perform exactly the same function, i.e., the innate CST does not activate afferent fibers, while the corticospinal interface does. Conversely, the activation of afferent fibers has a strong impact on the spinal circuits, which in turn generate a powerful motor output, thereby boosting the weakened motor output after SCI. Another difference is the delay of muscle activation. The latency of muscle activation from spikes of the linked neurons *via* the corticospinal interface (ave. ± s.d.: 53.7 ± 17.5 ms, range: 10–124 ms) was longer than that of innate corticospinal neurons innervating the forearm muscles of monkeys (3–18 ms) (Fetz and Cheney, 1980). The reason for the longer delay *via* the corticospinal interface might be because a 50-ms time window was used to average the firing rates of the linked neurons to achieve smoother changes in the stimulus parameters. Such a long latency may be solvable by improving the computational performance of the interface.

In our study, task performance in conjunction with the corticospinal interface was similarly achieved irrespective of the original firing patterns of the linked neurons before the corticospinal interface trials (Figure 4B). This indicates that the modulation of linked neurons is flexible and might be to some degree independent of their original firing patterns, which is consistent with previous studies demonstrating flexibility in controlling the firing rates of M1 cells (Fetz, 1969; Moritz et al., 2008). Thus, the corticospinal interface enabled the direct control of residual spinal circuits connected to the linked neurons and triggered the modulation of their firing pattern to regain impaired motor function after SCI.

Brain-controlled functional electrical stimulation of muscles can be used to control the magnitude of the stimulus-induced forces in a paralyzed upper limb (Moritz et al., 2008; Pohlmeyer et al., 2009; Kato et al., 2019). However, muscle stimulation activates the motor end plates or muscle fibers directly. Hence, muscular contraction is accomplished with an inverted recruitment order in which large diameter muscle fibers are activated preferentially, which is the opposite order from the physiological condition, thereby preventing smooth force control (McNeal and Reswick, 1976). In contrast, spinal stimulation recruits motoneurons *trans*-synaptically *via* afferent fibers (Mushahwar and Horch, 2000; Aoyagi et al., 2004; Bamford et al., 2005; Gaunt et al., 2006; Kato et al., 2019; Greiner et al., 2021; Kaneshige et al., 2022), so that motoneurons are activated in the natural order (Henneman, 1957; Henneman et al., 1965), which, in turn, may produce graded muscle contractions. Furthermore, spinal stimulation simultaneously activates excitatory and inhibitory interneurons to motoneurons (Nishimura et al., 2013; Guiho et al., 2021; Kaneshige et al., 2022) in the flexor and extensor muscles (Moritz et al., 2007; Nishimura et al., 2013; Greiner et al., 2021; Kaneshige et al., 2022), suggesting brain-controlled spinal stimulation *via* the corticospinal interface modulates force output by a similar mechanism that is closer to the physiological condition than *via* muscle stimulation.

### 4.3. Unlinked neurons

We previously demonstrated that closed-loop muscle stimulation using cortical oscillations induces targeted spatial changes in cortical activity in extensive areas. The strongest modulation of high-gamma activity became localized around an arbitrarily selected cortical site that controls stimulation (Kato et al., 2019). Although cortical oscillations, such as high-gamma activity, are thought to reflect the activity of neural assemblies in regions neighboring the recording site, it remains unclear how the neuronal activity of individual neurons is changed to incorporate the neural interface. Since we used a multi-electrode array, which allowed us to record assemblies of M1 neurons, we investigated how the unlinked neurons, which were not connected to the interface, modulated their activity in response to the corticospinal interface.

We found three types of unlinked neurons: “task-unrelated”, “increased”, and “decreased.” The firing rates of the “increased” and “decreased” unlinked neurons were modulated similarly to the linked neurons according to the required magnitude of wrist torque (Figure 7A). Since the activity of the “increased” unlinked neurons was associated with the activity of the linked neurons, they might have similar functions, e.g., they have similar preferred directions and/or receive a common upstream input. The activity of the “decreased” unlinked neurons showed the opposite activity pattern to the linked and “increased” unlinked neurons, which may indicate that there is reciprocal innervation between “decreased” unlinked neurons and a subgroup of linked neurons and “increased” unlinked neurons. Some “task-unrelated” unlinked neurons changed their firing characteristics to those of “task-related” neurons and became either “increased” or “decreased” neurons according to the demands of the task, i.e., weak or strong torque (Figures 7F, 8B, H). These subpopulations have presumably higher thresholds and receive common inputs with subpopulations that already exhibit either “increasing” or “decreasing” activity when weak torque is required.

The modulation of the unlinked neurons during the catch trials tended to be smaller than during the corticospinal interface trials (Catch in Figures 5A, 6B, H). This result suggests that many “task-related” neurons were affected by spinal cord stimulation *via* projections from the preserved ascending pathway, leading to the increased modulation of their activity during the corticospinal interface trials.

### 4.4. Clinical perspective and prospect

Of those people who survive a stroke, only 40–70% regain upper limb dexterity (Houwink et al., 2013). The major challenge in the field of neuroprosthetics is to restore dexterous finger movements and functionally coordinated multi-joint movements. The use of brain-controlled functional electrical stimulation of muscles should be effective in such cases, and previous studies have shown the restoration of a series of functional goal-directed limb movements (Ethier et al., 2012; Bouton et al., 2016). However, to induce functional movement of multiple joints, many electrodes must be implanted into many muscles. In contrast, spinal stimulation with a single electrode on the cervical cord evokes facilitative or suppressive responses in multiple muscles, including those located on proximal and distal joints, and activates synergistic muscle groups. For example, stimulation strongly facilitates finger flexor muscles, while it suppresses the antagonist muscles, which leads to coordinated movements similar to natural voluntary movements (Nishimura et al., 2013; Kato et al., 2019). Spinal stimulation may be a suitable target for restoring natural limb movements such as dexterous finger control and coordinated multi-joint movements of the hand-arm-shoulder. Since we used a single signal derived from the M1 to control stimulation of a spinal site, the degree of movement control demonstrated here remains limited (Figures 1, 3). Extending our paradigm to the control of more natural and complex movements would require additional input signals from unlinked neurons including increased, decreased, and unrelated types, and output to multiple spinal sites on rostral-caudal placements as well as ventral-dorsal placements of the spinal cord.

Since a substantial portion of the dorsal column sending somatosensory information upstream was lesioned in our SCI model (Figure 1C), the somatosensory function of the limb on the lesioned side seemed to be impaired (see section “3.1. A primate spinal lesion model”) and the monkeys might not have used somatosensory information for torque control. In the present study, the monkeys obtained visual feedback about the produced torque, suggesting that visual feedback might have compensated for the lost proprioceptive feedback. Actually, the monkeys were over-trained to perform the same task before SCI, and showed better task performance than with the corticospinal interface after SCI (see section “3.3. Volitional control of the paralyzed forearm *via* a corticospinal interface”), indicating that the associations between residual functions such as the level of effort required to exert torque and visual feedback of the exerted torque had already been well-established and might have been maintained even after SCI.

Somatosensory feedback is essential for the efficient and accurate control of force output and object manipulation. SCI and stroke commonly cause somatosensory dysfunction in addition to motor dysfunction. However, no therapeutic treatment for somatosensory dysfunction exists. Prior work has shown that direct cortical stimulation of the primary somatosensory cortex induces an artificial somatosensory perception according to somatotopy. Furthermore, there is a linear relationship between current intensity and the perceived intensity of the evoked sensation (Johnson et al., 2013; Hiremath et al., 2017; Lee et al., 2018; Kirin et al., 2019). These results suggest that the modulation of stimulation parameters such as current intensity and frequency to the primary somatosensory cortex can provide somatosensory feedback for tactile information and contact force in real time. The possibility of closing the loop for a bidirectional sensory-motor neuroprosthesis by coupling stimulation-evoked somatosensory feedback with real-time brain control of a paralyzed hand should be investigated in a future study.

## Data availability statement

The raw data supporting the conclusions of this article will be made available by the authors, without undue reservation.

## Ethics statement

This animal study was reviewed and approved by the Institutional Animal Care and Use Committee of the Tokyo Metropolitan Institute of Medical Science.

## Author contributions

KO and YN conceived and designed the experiment. KO, MK, MS, and YN performed the surgeries. KO conducted the experiments. KO, OY, and MK analyzed the results. KO, MK, TT, and YN wrote the manuscript. All authors read and approved the final version of the manuscript and agreed to be accountable for all aspects of the work in ensuring that questions related to the accuracy or integrity of any part of the work are appropriately investigated and resolved.
